# Effect of Osmotic Dehydration Pretreatment on the Drying Characteristics and Quality Properties of Semi-Dried (Intermediate) Kumquat (*Citrus japonica*) Slices by Vacuum Dryer

**DOI:** 10.3390/foods11142139

**Published:** 2022-07-19

**Authors:** Azime Özkan-Karabacak, Gülşah Özcan-Sinir, Ali Eren Çopur, Murat Bayizit

**Affiliations:** 1 Food Technology Program, Gemlik Asim Kocabiyik Vocational School, Bursa Uludag University, Bursa 16600, Turkey; 2 Science and Technology Application and Research Center (BITUAM), Bursa Uludağ University, Bursa 16059, Turkey; 3 Department of Food Engineering, Faculty of Agriculture, Bursa Uludağ University, Bursa 16059, Turkey; gulsahozcan@uludag.edu.tr; 4 Department of Agricultural Economics, Faculty of Agriculture, Bursa Uludağ University, Bursa 16059, Turkey; aerencopur@gmail.com (A.E.Ç.); mbayizit@gmail.com (M.B.)

**Keywords:** kumquat, osmotic dehydration, vacuum drying, drying characteristics, antioxidant capacity, total phenolics

## Abstract

The effect of osmotic dehydration (OD) pretreatments at different temperatures and immersion times on drying characteristics, total phenolic content (TPC), total antioxidant activity (TAA) (DPPH and CUPRAC methods), and color of kumquat slices dried under vacuum conditions (70 °C-100 mbar) was investigated. The OD pretreatment was performed in a sucrose solution (45 °Bx) at the temperatures of 40 and 50 °C and immersed at times of 30, 60, and 90 min. OD before vacuum drying decreased the total required drying time by up to 70 min compared to the control non-pretreated samples. Page, Modified Page, Henderson Pabis, and Two Terms Exponential models were found to satisfactorily describe the drying behavior of thin layer dried kumquat slices. The minimum and maximum values of effective moisture diffusivity (*Deff*) for semi-dried kumquat slices were 5.04 × 10^−8^ to 7.19 × 10^−8^, respectively. OD treatments induced a decline in TPC (5.30–33.92%) and TAA (23.63–59.34% and 4.17–31.67% for DPPH and CUPRAC assays, respectively) of kumquat slices. It was observed that OD pre-treatment can decrease the gross drying time, and make the color and sensorial attributes of dried kumquats better.

## 1. Introduction

Kumquat (*Citrus japonica*), the smallest of the true citrus fruits, is one of the citrus fruits eaten together with the peel, and has a sweet rind and an acidic pulp. Even though being native to South Asia and Asia-Pacific, kumquat trees, and evergreen shrubs, have been grown worldwide mainly as ornamental park and dooryard trees [1]. Though kumquat cultivations are spread to the Eastern Black Sea, Aegean, and Mediterranean Regions, it is still a little-known and consumed fruit in Turkey [2,3]. The world’s total citrus production is estimated at 158,490,986 tonnes for 2020, with China being the top producer, where about 10% was shared by the seedless pomelos, kumquats, and other minor citrus fruits [4].

Kumquat fruits are known to be an excellent source of nutrients and phytochemicals, such as ascorbic acid, carotenoids, flavonoids, phenolic compounds, minerals, and vitamins, present both in peel and flesh [5,6,7,8,9,10]. Due to their high bioactivity and nutritional value consumers prefer fresh kumquats, however, they can be consumed in processed forms (i.e., jam, marmalade, candy, beverages, liqueurs, or pickles) [11,12,13,14].

It is a well-known fact that adequate intake of fruits and vegetables has been related to protective benefits against several non-communicable diseases, such as the development of coronary heart disease, hypertension, and chronic obstructive pulmonary disease. However, because of the inherently perishable nature of the produce/short shelf life of these crops, as much as 30–35% of fruits and vegetables perish during harvest, storage, grading, transport, packaging, and distribution [15]. Besides their perishability, another barrier to increasing fruit and vegetable consumption is the time required to prepare them, which is understandable especially since convenience is one of the top global trends [16,17]. Thus, it is not surprising that if it comes to fruit, consumers require products available in many outlets most of the year, suitable for many uses, with a long shelf-life, and not messy [18]. At this point, the fruit industry is trying to meet the market demand for new, useful, and healthy products with fresh food characteristics and longer shelf life. To preserve fruits and vegetables for later use, several methods, i.e., canning, freezing, drying, fermentation, etc., can be applied. Drying, defined as the removal of water from solids through heat and mass transfer [9] has been referred to as a simple, safe, and convenient method with a high potential market, and dried foods are one of the commonly preferred forms of fruits as a healthy snack. Dried foods may be consumed all year through and their low moisture activity lets them keep longer than fresh food. The exploitation of dried fruit as a carrier of functional ingredients is a relatively new concept, although the functional properties of such products originated from the nature of the drying process, where the removal of water leads to a natural concentration of healthy fruit components [19].

However, due to longer drying times and higher drying temperatures, conventional drying processes cause the obtainment of products that have lower nutritional and sensorial features. Semi-dried (intermediate) foods which have very similar characteristics (color, texture, and flavor) to fresh foods, have achieved more interest in the global market [20]. Semi-dried foods have a characteristic moisture content between 20% and 30% and water activity between 0.70 and 0.85 [21]. Although the products in this water activity are considered microbiologically stable at room temperature, with the development of new packaging methods, longer shelf life can be obtained for semi-dried foods [20].

Citrus fruits can be dried by using convective drying, microwave drying, vacuum drying, and some other integrated techniques depending on their characteristics [10]. Conventional drying methods require more time and energy than combined or innovative techniques. Also, they can cause alterations in final goods such as tissue shrinkage, color, taste, and aroma shifts in a negative way with nutrition losses [11,12]. Prior to drying, several pre-treatments can be used to produce an intermediate moisture product and, as a result, improve the drying process and the end product quality with reduced drying time as well [13]. Osmotic dehydration (OD) is considered one of the best pre-treatments for reducing energy consumption, limiting thermal damage to products and increasing the effectiveness of drying. It comprises dipping the food material into a hypertonic solution, which results in the loss of water and small components from the test matrix to the osmotic solution, as well as the absorption of solid from the osmotic solution into the solid sample according to osmotic pressure differences. The density of mass transfer relies upon the type of osmotic agent, temperature, level of solute, stirring speed, dimensions of the fruit, ripeness level of fruits, and fruit to osmotic agent mass ratio. It is possible to use different osmotic solutes in the food industry such as; sucrose, glucose, fructose, maltodextrin, sorbitol, sodium chloride, and their mixtures [14,22,23,24]. OD has been applied as a pre-processing step in fruits like lemon [25], kiwi [26], pineapple [27], and apricot [28]. This pre-treatment is usually conducted at mild temperatures [29]. In these studies, the advantages of OD pretreatment were stated as that it requires low temperature and energy, inhibits browning by enzymes, and thus provides better retention of color and flavor of the food products, and reduces water activity.

Thin-layer drying models are important applications, which help with decisions involving the most suitable food-specific techniques, and predict and improve the dryers’ performances. The application of these equations allows the computing of the process limits as a function of time at any specific point in the dryer [30]. Among the drying techniques, vacuum drying is a method that is applied for the drying process of several agricultural crops, maintaining their color and nutrients [31]. This method is effective particularly for delicate foods because of faster drying, lower process temperatures, less shrinkage of the food, lacking of oxygen in the drying chamber, and less energy utilization [32,33]. Vacuum upgrades the mass exchange because of an expanded pressure gradient between within and outside of the specimen to dry and keeps a low-temperature level fundamental for heat-sensitive foods [34]. However, a limited number of studies have been found in the literature on drying kumquat [3,5,35]. To the best of my knowledge, there are no studies about OD pretreatment to obtain semi-dried kumquat slices. In these studies, mostly hot air and microwave drying methods were used. In our previous study [36], the effects of vacuum microwave and hot air methods on the quality parameters of kumquat slices were investigated. Although the drying time is shorter in the microwave and hot air drying methods compared to the vacuum drying method, it has been determined that the quality properties of the product are better preserved in vacuum drying [36]. Therefore, in this study vacuum drying was preferred as the drying method.

In this study, the drying aspects of the joint use of OD pre-treatment (40 and 50 °C) as a factor of soaking time (30, 60, and 90 min) and vacuum drying (70 °C at 100 mbar), as well as several quality properties of kumquat slices, were criticized. The obtained product was ready for consumption and can be qualified as an alternative functional product for the healthy snack sector.

## 2. Materials and Methods

### 2.1. Chemicals

All reagents used in this study were selected as pure for analytical analysis. Folin–Ciocalteu reagent, neocuproine (2,9-dimethyl-1,10-phenanthroline), DPPH (2,2-diphenyl-2-picrylhydrazyl), gallic acid, Trolox (6-hydroxy-2,5,7,8-tetramethylchroman-2-carboxylic acid) were purchased from Sigma Aldrich (Darmstadt, Germany). Methanol and sodium carbonate were obtained from Merck (Darmstadt, Germany).

### 2.2. Materials

Fresh kumquats (*Citrus japonica*, Fortunella japonica Swingle) were collected from a commercial garden in Antalya, Turkey, and were refrigerated at 4 ± 0.5 °C until the analysis. Before the drying process, kumquats were selected (with an average diameter 20.00 ± 0.25 mm), washed, and sliced (the thickness of 4.00 ± 0.08 mm). The initial moisture content of the kumquats was determined as 3.01 g water/g dry weight (dw) by using an infrared moisture analyzer (Sartorius MA150, Sartorius AG, Göttingen, Germany).

### 2.3. Osmotic Dehydration

The kumquats were immersed in the osmotic solution made of sucrose (45° Bx) at 40 and 50 °C. The beakers with kumquat samples (50 g) and osmotic solution (200 g) were placed into water bath (Memmert, WNE14, Germany). Water bath (static) and osmotic solutions were set to the selected temperatures 30 min before the start of the experiment. Later, osmotic pretreatment was applied for 30, 60, and 90 min by immersing the sliced kumquats. When the immersion time was reached, kumquats were collected from solution and slightly dried with an absorbent paper to eliminate excess solution.

### 2.4. Vacuum Drying

Drying was carried out in a vacuum dryer (Memmert, VO400, Schwabach, Germany, 49 L volume) at a temperature of 70 °C with vacuum pressures of 100 mbar. In our previous study we researched different drying methods and conditions for drying kumquat slices and vacuum drying at 70 °C and 100 mbar provided excellent results [36]. For this reason, this parameter was chosen for drying of OD treated and semi-dried kumquat slices. A total of 50 g of samples were placed on the square aluminum plate and located in the shelves of vacuum dryer. The moisture loss of the samples during drying was measured using a digital balance (Mettler Toledo, MS3002S, Columbus, OH, USA) with 0.01 g precision and recorded at 10 min intervals for 1 to 2 h according to drying performance of the samples. All drying experiments were carried out with 3 replications and final moisture content of all dried samples was recorded as 0.55 g H_2_O/g dry weight (dw).

### 2.5. Mathematical Modelling of Drying Kinetics

Seven thin layer drying models used for describing OD-treated kumquats drying data are given in the following Equations [10]:

Page; MR = *exp(−kt^n^)*, (1)

Modified Page; MR = *exp [(−kt)^n^]*, (2)

Logarithmic; MR = *a exp(−kt) + c*, (3)

Lewis; MR = *exp(−kt),* (4)

Henderson & Pabis; MR = *a exp(−kt)*, (5)

Two Term Exponential; MR = *a exp(−kt)* + (1 − *a*) *exp(−kat)*, (6)

Wang & Singh; MR = 1 + *at* + *bt*^2^, (7)

In these equations; *a*, *b*, *c*, and *n* symbolize model constant, *k* represents model coefficient (1/s) and, *t* denotes the drying time (s).

In the modeling, Equation (8) was used to calculate the moisture ratio (*MR*).

(8) $$MR=\frac{M-M_{e}}{M_{i}-M_{e}}$$

In the formula, *M* is the moisture content at a specific time (g water/g dw), *M_i_* is the moisture content of the sample prior to drying (g water/g dw) and *M_e_* is the equilibrium moisture content (g water/g dw). *M_e_* is relatively small compared to *M* or *M_i_* values, and hence can be neglected.

In the determination of the best model, Chi-square (χ2), root mean square error (*RMSE*), and correlation coefficient (*R*^2^) statistical criteria were used and given in the following equations:

(9) RMSE=[1N∑i=1N(MRexp,i−MRpre,i)2]12

(10) $${}^{\chi 2}=\frac{\sum_{i=1}^{N}{\left(MR_{\exp,i}-MR_{pre,i}\right)}^{2}}{N-n}$$

In the above equations; *MR_exp,i_* and *MR_pre,i_* represent the moisture ratios of experimental and dimensionless, respectively for the test *i*. *N* and *n* are the number of observations and model constant number, respectively.

### 2.6. Effective Moisture Diffusivity

Assuming that in the drying process of kumquat slices, the diffusion coefficient is constant, moisture change is resulted only by diffusion, shrinkage is negligible, initial moisture concentration is uniform and the samples are considered as infinite slab geometry, the Effective Moisture Diffusivity (*D_eff_*) can be obtained through Equation (11) [37]:

(11) $$MR=\frac{8}{\pi^{2}}\sum_{n=1}^{\infty }\frac{1}{{\left(2n-1\right)}^{2}}\exp\left(-\frac{{\left(2n-1\right)}^{2}\pi^{2}D_{eff}t}{4L^{2}}\right)$$

where, *D_eff_*, L and n represent effective moisture diffusivity (m^2^/s), half thickness of the slab in samples (m), and a positive integer, respectively. After simplification by drawing log graphs of the acquired data versus time, Equation (12) is obtained.

(12) $$D_{eff}=-\frac{slope4L^{2}}{\pi^{2}}$$

### 2.7. Color Analysis

The colors of fresh, non-pretreated and OD-treated kumquats were measured with a Hunter Lab MiniScan EZ4500L spectrophotometer. The instrument has a 45°/0° geometry with a directional annular 45° illumination and a 0° viewing (specular components excluded). The colors of fresh, non-pretreated and OD-treated kumquats were measured with a colorimeter (Hunter Lab MiniScan, EZ4500L, Reston, VA, USA). Before color measurements, the instrument was calibrated with black and white ceramic plates. L*, +a*, −a*, +b* and −b* values indicate the color brightness (changed from 0 = black to 100 = white), redness, greenness, yellowness and blueness, respectively. In addition, Chroma (C*) which represents color intensity, and hue angle (h°) denotes color changes with the angles (0° or 360° = red, 270° = blue 180° = green and 90° = yellow) were calculated by using L*, a* and b* values by using the following equations:

(13) $$C^{*}=\sqrt{{\left(a^{*}\right)}^{2}+{\left(b^{*}\right)}^{2}}$$

(14) $$h^{o}=arctan\;\left(\frac{b^{*}}{a^{*}}\right)\;$$

### 2.8. Preparation of Extracts for Total Phenolic Content and Antioxidant Capacity

The extracts of fresh, non-treated and OD-treated kumquats were processed in line with Vitali et al. [38]’s recommendations with slight modifications. Briefly, 2 g of kumquat samples pestled and mixed with 20 mL extraction solution containing HCl: water: methanol with the ratios of 1:10:80 *v*/*v*. After the mixture was shaken at 250 rpm for 2 h at 20 °C (JB50-D rotary shaker, Shanghai Shengke Instruments, Shanghai, China), it was centrifuged at 3500 rpm for 10 min (Sigma centrifuge 3K 30, Osterode am Harz, Germany). Obtained extracts were stored at −20 °C until analysis.

### 2.9. Determination of TPC and TAA

Folin-Ciocalteu spectrophotometric methodology stated by Spanos and Wrolstad [39] was used to determine TPC with slight modifications. The results were given in mg of gallic acid equivalent (GAE) per 100 g dw of sample. 

TAA of the semi-dried kumquat slices were performed according to 2-diphenyl-1-picrylhydrazyl (DPPH) and Copper (II) reducing antioxidant capacity (CUPRAC) methods according to Katalinic et al. [40] and Apak et al. [41], respectively. In both assays, the results were expressed in terms of μmol Trolox equivalent (TE) per 1 g dw.

### 2.10. Sensory Analysis

Color, appearance, taste, chewiness, and general acceptability of semi-dried kumquat slices were evaluated by nine trained panelists. These panelists were chosen among academicians and graduate students in Bursa Uludag University Food Engineering Department. A nine-point hedonic scale which scale changed from “like extremely (9)” to “dislike extremely (1)” was applied. Randomly coded kumquat samples were served to the panelists.

### 2.11. Statistical Analysis

All experimental measurements were performed with three replicates. The results were statistically calculated by analysis of variance (ANOVA) using SPSS for Windows (Version 23). When significant differences were found (*p* < 0.05), the DUNCAN multiple range test was utilized to define the differences among means. 

## 3. Results and Discussion

### 3.1. Drying Kinetics of Kumquat Slices

**Figure 1:**
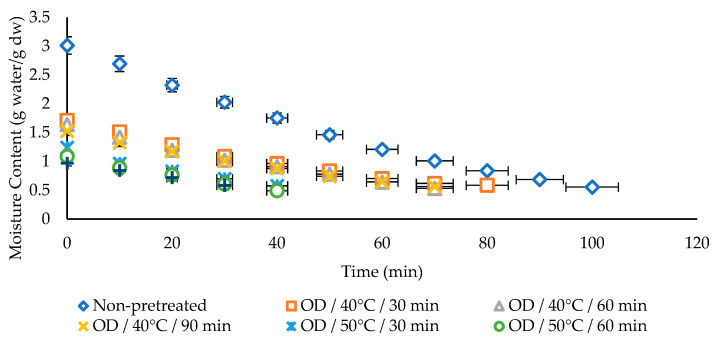
Drying behaviors of kumquat slices.

The differences in moisture content against drying time for non-pretreated and OD- treated kumquat slices were shown in Figure 1. The initial moisture content of the samples was affected by the OD temperature and time and was obtained as follows, from highest to lowest; non-pretreated, OD/40 °C/30 min, OD/40 °C/60 min, OD/40 °C/90 min, OD/50 °C/30 min, OD/50 °C/60 min, and OD/50 °C/90 min. The drying time was shortened as the initial moisture content decreased, under OD applied conditions. While the longest drying time was obtained by non-pretreated samples, osmotically dehydrated samples at the higher temperature and the longer application time (OD/50 °C/90 min) showed the shortest drying time. The experimental results showed that drying time was reduced by increasing the temperature and application time of OD for kumquat slices. Moreover, OD treatment shortened the drying time between 20% and 70% compared to non-pretreated samples. However, the initial moisture content is different, there was no change in drying time between OD/50 °C/30 min and OD/50 °C/60 min and between OD/40 °C/60 min and OD/40 °C/90 min applications.

Our results are close to those observed by Sakooei-Vayghan et al. [14] where OD pretreatment before hot-air drying for 30 and 45 min decreased drying time to 8 and 7 h, respectively for apricot cubes. Moreover, Bchir et al. [42] emphasized that ultrasound-assisted OD reduced the drying time of pomegranate seeds by over 40%. These results showed parallelism with our research. 

In the literature, it is also stated that using OD with vacuum drying gets the quality of the food products much better than that of food products produced by OD alone. This situation explained such combining vacuum drying with OD-facilitated penetration of osmotic solutions into porous structures of food tissues in a controlled manner [20].

### 3.2. Modeling of Drying Data

The statistical analysis results for the seven different models used for the values obtained from the osmotic pre-treated kumquat slices are given in Table 1. The lowest RMSE and χ2 values and the highest R^2^ values were deemed to be the suitable model. Page and Modified Page are the appropriate models for the non-pretreated samples and OD/40 °C/30 min, OD/40 °C/60 min, OD/50 °C/90 min pre-treated kumquat slices. However both the Henderson Pabis and Two Terms Exponential models best describe the moisture content data of the OD/40 °C/90 min, OD/50 °C/30 min, and OD/50 °C/60 min pre-treated kumquat samples according to the statistical parameters. For the best models, the R^2^, RMSE, and χ2 values are mean in good fit ranging from 0.9925–0.9994, 0.001590–0.005885, 0.000014–0.000269, respectively. 

Similar findings were achieved by various researchers for the models of Page and Modified Page such as Sobukola [43] and Kumar et al. [44] on okra and onion, respectively. da Cunha et al. [45] found the Two Terms Exponential model as the most appropriate one for melon. The Henderson and Pabis model for orange [46] was also decided as the most proper theoretical model in the literature. 

In our previous study, we found Page and Modified Page models as best fitted models for kumquat slices dried by microwave, hot air, and vacuum drying methods [36].

### 3.3. Effective Moisture Diffusivity (Deff)

**Figure 2:**
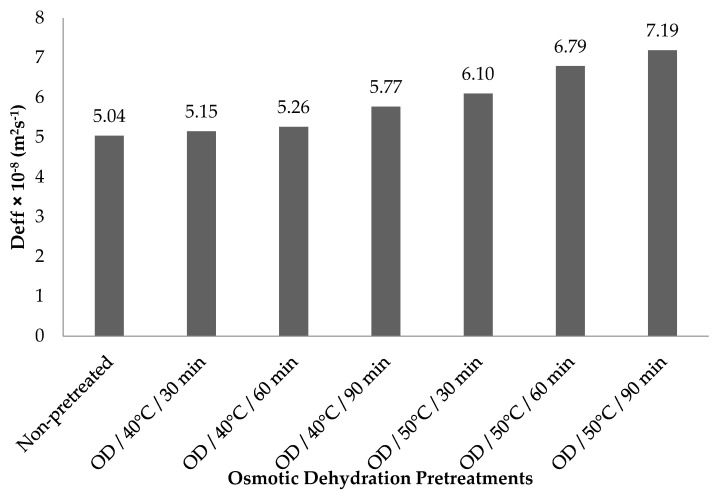
The estimated *Deff* values of semi-dried kumquat slices.

The estimated *Deff* values for all pretreatments are shown in Figure 2. The *Deff* values for different pretreatments, ranged from 5.04 × 10^−8^ to 7.19 × 10^−8^ in non-pretreated control samples and OD/50 °C/90 min, respectively. Our *D_eff_* results were consistent with the general range (10^−12^–10^−8^ m^2·^s^−1^) for agricultural materials [47]. When compared with non-pretreated control, osmotically dehydrated kumquat samples showed higher *Deff* values. The increments in *Deff* values may be explained by the less ‘‘case-hardening’’ impact of samples after osmotic dehydration, which led to increased evaporated transition [48]. The results demonstrate that the rise in temperature and time of OD pretreatment brings about an increment in the value of *Deff*. Higher OD temperatures and times lead to higher heating energy and thus water molecules with increased activity cause high moisture diffusion [49]. Consistent with our results, An et al. [48] also reported that the *Deff* value of osmotically dehydrated cherry tomatoes was higher than that of fresh samples at the end of hot air drying.

### 3.4. Total Phenolic Content (TPC)

**Figure 3:**
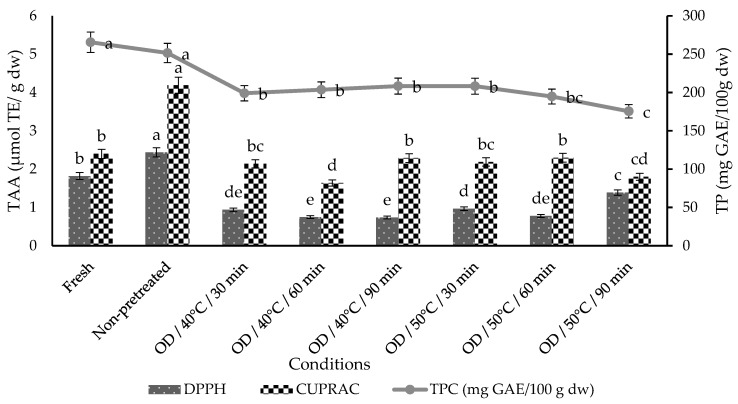
The effect of drying treatments on kumquats’ TAA and TPC. Different lower letters in bars and lines display significant differences (*p* < 0.05), GAE: gallic acid equivalent, TE: trolox equivalent, dw: dry weight.

The TPC availability in fresh, non-pretreated, and pretreated with osmotically dried kumquat slices was given in Figure 3. TPC of fresh kumquat was 265.62 ± 12.41 mg GA/100 g dw. The TPC of fresh kumquat slices was in line with our previous results for kumquat slices as 266.68 ± 14.57 mg GA/100 g d.w. [36]. 

TPC of kumquat slices decreased after vacuum drying between 5.30–33.92% for non-pretreated and OD/50 °C/90 min pretreated kumquat samples. The decrease in TPC in kumquat samples with drying is explained by the fact that polyphenols are not heat-stable and long-term heat applications may create permanent chemistry-based alterations in these compounds [50,51]. Moreover, this reduction can be associated with the binding of polyphenols with other compounds (proteins) or the chemical structure of polyphenols that cannot be extracted or determined by current methods [50]. In addition, activation of oxidative enzymes such as polyphenol oxidase and peroxidase is another important factor related to the loss in TPC [52,53]. Similar results have also been reported by Ozkan-Karabacak et al. [10], Yu et al. [54], and Turkiewicz et al. [55] in which TPCs were decreased after drying. 

The present study showed that OD pretreatment with different temperatures and times had a significant decrement in the TPC when compared to non-pretreated control (*p* < 0.05). After drying, the greatest loss in TPC (33.92%) was observed at the highest temperature and time (50 °C/90 min), while a temperature of 40 °C led to a loss between 21.57–25.07%. The smallest losses (5.30%) were observed in non-pretreated control samples. These findings are consistent with the results conducted by Kucner et al. [56]. This result can be explained by phenolic components migrating faster to the OD solution due to the increased temperature. This can be explained by increased temperature causing more phenolic compounds to migrate to the dehydrating solution. A rise in temperature causes an increase in the diffusion flow rate, and the selectivity of cell membranes is also inhibited by high temperature [56]. In contrast with our study, Dermesonlouoglou et al. [57] found that goji berry fruits presented the highest TPC increasing with OD time and temperature.

### 3.5. Total Antioxidant Activity (TAA)

The TAA of the fresh and the vacuum dried kumquat slices were given in Figure 3. Fresh kumquat slices contain 1.82 and 2.40 µmol TE/g dw TAA in DPPH and CUPRAC assays, respectively. The final values of the CUPRAC method were found to be 1.32 times higher than the results of the DPPH method. This may be due to the fact that while both hydrophilic and hydrophobic antioxidants in foods can be detected with the CUPRAC method, only hydrophobic antioxidants can be detected with the DPPH method [10]. 

The TAA of the kumquat slices was reduced (between 23.63–59.34% for DPPH, 4.17–31.67% for CUPRAC) for osmotically pretreated kumquat slices whereas the increment (34% for DPPH, 74% for CUPRAC) was observed for non-pretreated kumquat slices. The lowest TAA values were obtained from the OD/40 °C/60 min pretreated kumquat slices for both the DPPH and CUPRAC assays in all osmotically pretreated samples. This decrease may be related to the impact of osmotic processes on mass transfer, because some of these components are considered water-soluble. A similar result regarding the decrease in TAA and TPC of papaya samples with osmotic pretreatment, was observed by Udomkun et al. [58]. However, the reason for the increment in TAA of dried kumquats without pretreatment, may be dependent on the formation and accumulation of Maillard reaction products (such as melanoidins) having an antioxidant capacity [59]. 

### 3.6. Color

One of the most critical factors influencing product quality and consumer preference is color. The color values of fresh and pre-treated vacuum-dried kumquat slices are shown in Table 2. L* value of fresh kumquat slices found as 61.04. L* value of kumquat slices was significantly decreased after drying (*p* < 0.05) and the highest decrement was observed at non-pretreated kumquat slices (55.06%). In osmotically pretreated samples, the significantly highest reduction was observed at 40 °C pretreated samples regardless of their times (*p* < 0.05). The decrease in the L* value, which represents the lightness, means a darker color. This behaviour might be related to the longer drying times at lower a temperature (40 °C) of osmotic solution (70–80 min) and non-pretreated samples (100 min). In OD applied at low temperatures, high-concentration sugar prevents discoloration as it covers fruit and vegetable parts. However, in the case of exceeding 45 °C, color changes occur in the fruit [60]. 

The values of a* representing redness (+) and greenness (−) varied between 11.82 and 18.30 (Table 2), and the highest value was observed at OD/50 °C/90 min treatment. The formation of color changes due to the Maillard reaction throughout the drying process is a decisive factor in the formation of red color. Color in fruits and vegetables depends on the presence of pigments such as anthocyanin, flavanol, chlorophyll, and carotene. Of these pigments, anthocyanins and flavanols are soluble in water, while carotene and chlorophylls are insoluble in water [60].

Compared with fresh samples, the b* value decreased afterward drying between 34.29 and 48.87% for OD/50 °C/30 min and OD/40 °C/30 min, respectively. To obtain a higher b* value it could be recommended to use a higher osmotic solution temperature and lower application time.

The Chroma (C*) is a measure of chromaticity that indicates the purity or saturation of a color. The C* values tend to decrease during the drying process. The lowest C* value of 37.57 was observed in kumquat slices which were subjected to OD pre-treatment at 40 °C-30 min. On the other hand, *h°* values are examined, the highest value is found at 75.63 in fresh kumquat slices, while the lowest value belongs to the kumquat slices that are vacuum dried after being subjected to OD pre-treatment at 50 °C/90 min. The decrement in *h°* values demonstrated the darkening of kumquat slices.

Cháfer et al. [61] investigated developing new minimally processed citrus peel products from orange peels and one of the quality criteria they want to protect was color. They analyzed color coordinates (L*, C*, and *h°*) of fresh, osmotic dehydrated (OD) and vacuum pulsed osmotic dehydrated (VPOD) orange peels. L* values were determined as 67, 66, and 58 flavedo layers of the orange peel of fresh, OD, and VPOD, respectively, while C* values were 78, 76, and 61, and *h°* values were 64, 64, and 65.

### 3.7. Sensory Analysis

**Figure 4:**
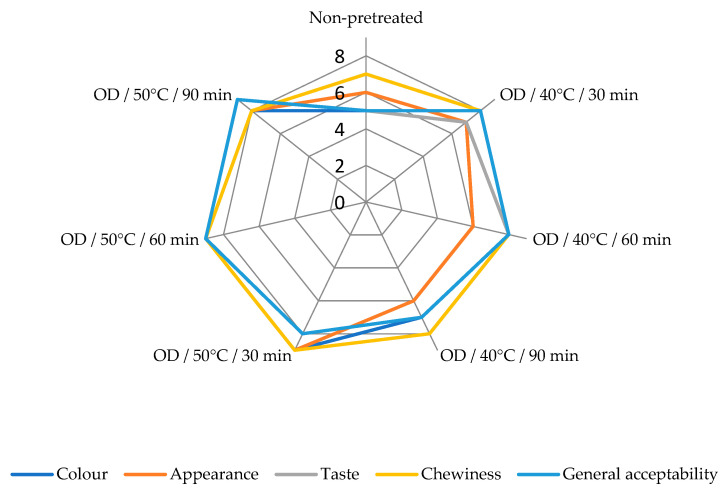
Sensory properties of dried kumquat slices.

Semi-dried kumquat slices were organoleptically assessed for their color, appearance, taste, chewiness, and general acceptability (Figure 4). Significant differences in sensorial properties of kumquat slices were observed (*p* < 0.05). The most liked product by the panelists was OD pretreated at 50 °C. The pretreatment of OD/50 °C/60 min got the highest score in terms of all sensory parameters when compared to other pretreatments and non-pretreated control. Non-pretreated kumquat slices were less preferred than OD pretreated samples. The greater acceptance of the OD pretreated samples’ taste may be related to the suppression of the bitter taste that may come from the peels of the kumquat slices, thanks to the amount of sugar transferred from the OD solution to the product.

## 4. Conclusions

The current study explained the significance of OD pretreatment on drying kinetics, and some quality attributes of vacuum-dried kumquats. The Page, Modified Page, Henderson Pabis, and Two Terms Exponential models provided a strong statistical fit for drying kumquat slices. The higher L* values were observed from osmotically pretreated kumquats when compared with non-pretreated control. The *Deff* value of kumquat slices increased when the temperature and time of the osmotic solution were raised. Also, osmotically dehydrated kumquat slices showed higher *Deff* values than non-pretreated control. OD pretreatment at 50 °C got the highest score from the panelists in terms of sensory properties. However, TPC and TAA of vacuum-dried kumquat slices were reduced in comparison with raw material.

In general, the application of OD shortened the vacuum drying time and increased the effectiveness of drying. From the obtained results, it is recommended to use 50 °C OD solution since the low influence on the quality parameters, lowest possible browning and lowest drying times of dried kumquats were observed. However, further research is still needed to fully optimize this combined drying treatment.

## Figures and Tables

**Table 1:** Statistical results obtained from the modeling of kumquat slices.

Model Name			Non- Pretreated	OD/40 °C/30 min	OD/40 °C/60 min	OD/40 °C/90 min	OD/50 °C/30 min	OD/50 °C/60 min	OD/50 °C/ 90 min
Page	Model coefficient	n	1.1754	1.0504	1.0326	1.0177	0.7871	0.9964	1.1563
		k	0.0074	0.0117	0.0136	0.0132	0.0404	0.0195	0.0097
	R^2^		0.9994	0.9942	0.9970	0.9990	0.9911	0.9906	0.9976
	RMSE		0.001590	0.004824	0.003423	0.001635	0.004578	0.005208	0.002672
	X^2^		0.000014	0.000269	0.000125	0.000029	0.000175	0.000226	0.000057
Modified Page	Model coefficient	n	1.1754	1.0504	1.0326	1.0177	0.7871	0.9964	1.1563
		k	0.0154	0.0145	0.0156	0.0142	0.0169	0.0192	0.0181
	R^2^		0.9994	0.9942	0.9970	0.9990	0.9911	0.9906	0.9976
	RMSE		0.001590	0.004824	0.003423	0.001635	0.004578	0.005208	0.002672
	X^2^		0.000015	0.000269	0.000125	0.000029	0.000175	0.000226	0.000057
Logarithmic	Model coefficient	k	0.0303	0.0464	0.0361	0.0393	0.0550	0.0530	0.0525
		a	1.0843	1.0411	1.2629	1.2825	1.8214	1.6706	2.4225
		c	0.1841	0.3429	0.3255	0.3688	0.4638	0.4520	0.6039
	R^2^		0.9373	0.8943	0.9473	0.9410	0.9891	0.9564	0.9818
	RMSE		0.028489	0.045496	0.092439	0.098741	0.313986	0.271615	0.623710
	X^2^		0.012275	0.027943	0.109375	0.124799	1.232343	0.922182	6.224232
Lewis	Model coefficient	k	0.0159	0.0142	0.0155	0.0142	0.0195	0.0194	0.0161
	R^2^		0.9871	0.9936	0.9965	0.9991	0.9884	0.9960	0.9895
	RMSE		0.009625	0.004251	0.003599	0.002097	0.009897	0.005198	0.007103
	X^2^		0.001121	0.000183	0.000118	0.000040	0.000612	0.000169	0.000269
Henderson & Pabis	Model coefficient	k	0.0171	0.0141	0.0158	0.0144	0.0186	0.0197	0.0167
		a	1.0846	1.0070	1.0105	1.0080	1.0296	1.0078	1.0151
	R^2^		0.9935	0.9937	0.9968	0.9993	0.9925	0.9962	0.9918
	RMSE		0.009228	0.004914	0.003767	0.001054	0.004310	0.005035	0.006627
	X^2^		0.001145	0.000279	0.000151	0.000015	0.000078	0.000217	0.000351
Two Term Exponential	Model coefficient	k	0.0112	0.0094	0.0105	0.0096	0.0123	0.0131	0.0111
		a	0.5203	0.5017	0.5026	0.5020	0.5073	0.5019	0.5037
	R^2^		0.9935	0.9937	0.9968	0.9993	0.9925	0.9962	0.9918
	RMSE		0.052050	0.062597	0.061812	0.003983	0.005885	0.002325	0.052887
	X^2^		0.036424	0.045341	0.040755	0.000186	0.000092	0.000159	0.022376
Wang & Singh	Model coefficient	b	0.00003	0.00006	0.00007	0.00002	0.0001	0.0001	0.0006
		a	−0.0117	−0.0131	−0.0144	−0.0134	−0.0241	−0.0188	0.0042
	R^2^		0.8900	0.8810	0.9347	0.9680	0.8786	0.8141	0.7374
	RMSE		0.006476	0.004524	0.004120	0.003151	0.012696	0.013371	0.246608
	X^2^		0.000564	0.000237	0.000181	0.000106	0.001343	0.001490	0.506795

**Table 2:** Color values of fresh and pre-treated and vacuum dried kumquat slices.

	L*	a*	b*	C*	*h°*
Fresh	61.04 ± 0.15 ^a^	16.27 ± 0.03 ^bc^	63.52 ± 0.18 ^a^	65.57 ± 0.17 ^a^	75.63 ± 0.06 ^a^
Non-pretreated	27.43 ± 1.22 ^e^	14.23 ± 1.05 ^de^	35.48 ± 0.62 ^cd^	38.24 ± 0.59 ^de^	68.15 ± 1.61 ^bc^
OD/40 °C/30 min	33.06 ± 0.41 ^cd^	11.82 ± 0.54 ^f^	32.48 ± 0.63 ^d^	34.57 ± 0.77 ^f^	70.01 ± 0.50 ^b^
OD/40 °C/60 min	32.62 ± 1.49 ^d^	16.52 ± 0.68 ^abc^	36.47 ± 3.82 ^cd^	40.06 ± 3.54 ^cd^	65.51 ± 2.32 ^cd^
OD/40 °C/90 min	30.70 ± 1.11 ^d^	15.38 ± 0.86 ^cd^	37.22 ± 0.79 ^c^	40.27 ± 1.05 ^cd^	67.56 ± 0.71 ^bcd^
OD/50 °C/30 min	36.35 ± 0.38 ^b^	17.50 ± 0.39 ^ab^	41.74 ± 0.51 ^b^	45.26 ± 0.62 ^b^	67.26 ± 0.21 ^bcd^
OD/50 °C/60 min	36.12 ± 1.67 ^b^	12.91 ± 0.08 ^ef^	35.20 ± 0.61 ^cd^	37.50 ± 0.59 ^de^	69.86 ± 0.22 ^b^
OD/50 °C/90 min	35.65 ± 0.22 ^bc^	18.30 ± 0.78 ^a^	39.47 ± 1.78 ^bc^	43.50 ± 1.94 ^bc^	65.11 ± 0.17 ^d^

^a–f^ Different letters in the same column display significant differences (*p* < 0.05).

## Data Availability

Data is contained within the article.
